# Efficacy Beliefs, Empowering Leadership, and Project Success in Public Research Centers: An Italian–Polish Study

**DOI:** 10.3390/ijerph18136763

**Published:** 2021-06-23

**Authors:** Guido Capaldo, Vincenza Capone, Jolanta Babiak, Beata Bajcar, Dorota Kuchta

**Affiliations:** 1 Department of Industrial Engineering, University of Naples Federico II, 83100 Naples, Italy; guido.capaldo@unina.it; 2 Department of Humanities, University of Naples Federico II, 83100 Naples, Italy; 3 Department of Management Systems and Organizational Development, Faculty of Computer Science and Management, Wrocław University of Science and Technology, 50-370 Wroclaw, Poland; jolanta.babiak@pwr.edu.pl (J.B.); beata.bajcar@pwr.edu.pl (B.B.); dorota.kuchta@pwr.edu.pl (D.K.)

**Keywords:** soft skills, project management, efficacy beliefs, project success, satisfaction with the project, public research

## Abstract

In the world of university research, although the figure of project manager is not formally foreseen, the principal researcher (PR) is, at many times, the last responsible the project results, schedule, and cost. The study aimed to investigate, in the light of the literature and through a cross-cultural study conducted in Italy and Poland, the relationship between soft skills (empowering leadership style, self-efficacy beliefs, and collective efficacy) of the principal researcher (PR) and the perceived success of research projects and satisfaction with the project, taking into account cross-cultural differences. A total of 67 PRs of complex projects in public universities (28 in Italy and 39 in Poland) participated in the study, completing a self-report questionnaire. Data were analyzed using descriptive and correlational analyses. The results showed a significantly higher mean value for team management self-efficacy in a Polish sample and a higher satisfaction with projects in Italian sample. All the soft skills included in the study were related to project success and satisfaction with the project. The results could be used to identify possible ways of intervention to establish a more mature project culture in public research organizations.

## 1. Introduction

In the literature on project management, the relationship between project leadership and project success has been highlighted by numerous researchers in various manufacturing and service sectors [1]. 

There are several studies on principal researchers (PRs) acting as project managers, but the majority of them concern cases of PRs working in industry or private research centers. As [2] highlighted, industry research projects differ significantly from pure academic research projects in how they are planned and managed. In academic contexts, contrary to industry ones, PRs often lack knowledge and skills related to project management systems, formal authority over the project team (they are “peers among peers”), and project administrative support [3,4].

As affirmed by Riol et al. [5], there appears to be no empirical documentation on the management of research projects by PRs in universities. Similarly, Fowler et al. [6] claimed there has been little debate concerning the development of project management in the academic context.

In recent years, budget cuts and limited funds in public universities have increased the number of projects funded in highly competitive calls and tenders. As a consequence, project management skills need to be enhanced in these settings to ensure research projects are managed successfully. In the world of academic research, although the figure of project manager is not formally appointed, the PR often has final responsibility for a project’s results, schedule, and cost. For these reasons, we could affirm that the PR is responsible for the management of the research project. The PR acts as the project manager for research projects even if this role is not formally acknowledged in public research bodies such as universities [7].

A research project can be defined as “the realization of a particular purpose, not always precisely worded, allowing new knowledge about the reality that surrounds us” [8] (p. 57). In Jordan et al. [9] we can find various classifications of research projects, among others, in two basic categories: (a) narrow scope of focus; small, autonomous projects; and (b) broad scope of focus; large, coordinated programs. In this paper, we focused on the latter. Following Ernø–Kjølhede [10], we took into account that certain adjustments are needed when referring to research project management in universities, compared to general project-based research settings. The “hard” or technical tenets of project management (e.g., divide the project into distinct phases and sub-tasks, set clear goals, emphasize planning and control, limit uncertainty, and avoid risks) only partially fit research peculiarities such as uncertainty of outcome and the difficulties of planning activities and measuring and monitoring results. This is clearly shown by Kuchta and Skowron [11], where the need for a special approach to research projects’ management is justified. In academic research domains, project management is often applied to individual and team-based tasks that involve high levels of creativity and flexibility. Fowler et al. [6] confirmed that research and development (R&D) project managers (and participants) in projects carried out by the Academy of Science, tertiary educational institutions, and research institutes adopted almost no project management methodology. (We understand project management methodology as strictly defined combinations of practices regarding logic, methods, and processes that determine how to plan, develop, and control a project along the continuous process of its implementation and successful completion [12].)

Based on the considerations above, our study assumed that the “soft” side of project management is extremely important for the research world, and especially for public research institutions. Soft skills, or people skills, enable the project manager to effectively interact both with team members and project stakeholders (i.e., all groups and individuals interested in or influenced by the project, including the members of the project team, the organization’s departments, sponsors, and potential beneficiaries; [13]).

Among the different typologies of soft skills, several authors have highlighted that leadership is essential in managing research projects. Researchers have found that autonomy-supportive leadership fosters better performance in academic settings [14]. The research project leader should create a cooperative climate that facilitates creativity and innovation and develops an atmosphere of excitement and commitment to the project [2,13,15,16]. In this context, empowering leadership is gaining attention (see [17] for a review), reflecting a current trend toward increased autonomy as a means of motivating employees. Empowering leadership focuses on actions such as enhancing meaningfulness of work, expressing confidence in performance, shared decision making, providing autonomy [18], and leading by example [19].

From a social cognitive perspective [20], when analyzing the relationship between the PR’s leadership and the success of research projects, it may be appropriate to consider the PR’s efficacy beliefs. Bandura [20] defined self-efficacy as “beliefs in one’s capabilities to organize and execute courses of action required in managing prospective situations. Self-efficacy has a strong influence on human behavior [20]. Individuals with greater perceived confidence with regard to a particular task, skill, or action may be more likely to engage in the behavior. Unless people believe they can produce desired effect by their actions, they have little incentive to act. Furthermore, high levels of self-efficacy have beneficial effects on individual functioning and satisfaction with work [21].

In public research settings, researchers have reported that self-efficacy beliefs are correlated with other self-beliefs, motivation constructs, and career choices, changes, and achievement, although effect sizes and relationships much depend on how self-efficacy and criterial tasks are operationalized and assessed [22]. Efficacy beliefs influence how people think, feel, motivate themselves, and act” (p. 2). These could be defined as efficiency-oriented competencies [23] and could represent an essential requirement for an academic project manager. 

A further consideration for research management in public institutions is team effectiveness. In modern organizational settings, teamwork is essential to fostering an environment that supports and enhances a high-quality, person-centered work culture [24]. From this point of view, team effectiveness is the capability of a research group to achieve team goals and objectives [25,26]. From a management perspective, [7] highlighted that team effectiveness could be considered a precondition for the success of R&D projects. Success levels are a difficult concept to deal with and usually only measure subsequent economic success, publications, or patents. 

It is appropriate to keep in mind the specificities of academic project teams, such as the peer-among-peers structure and the limited knowledge of project management methodologies. Bandura [20] introduced collective efficacy beliefs, which he defined as “a group’s shared belief in its conjoint capabilities to organize and execute the courses of action required to produce given levels of attainments” (p. 447). PR with high levels of collective efficacy referring to his/her group competencies could orient behavior towards the planning and use of shared resources and the willingness to persist, despite internal conflicts, changes in the political environment, or social concerns [19].

In the public research context, successful projects also need to effectively engage project partners, and a key question is how to adequately engage those from foreign cultural backgrounds. Cross-cultural project management is an important challenge for project managers and project management scholars, and the concepts of leadership and culture may be the most debated subjects in the management literature [27]. To date, we still know very little about the way national culture influences leadership styles in public research.

Defining project success is a complex issue [28,29] but it is generally accepted that, apart from objective criteria (budget, deadline etc.), the principal stakeholders’ satisfaction (and therefore the PR’s) has to be considered [30]. Based on the above considerations, our study aimed to provide answers to the following questions:

- What is the relationship between a PR’s empowering leadership (when acting as project manager of a research team) and the research project’s success?
- What is the relationship between efficacy beliefs and empowering leadership, project success, and the PR’s satisfaction with the project?

To date, we still know very little about the way national culture influences leadership styles in public research. 

Therefore, the specific purpose of our research was to examine the relationship between the PR’s skills (i.e., team management and project management self-efficacy, collective team efficacy as perceived by PR, and empowering leadership) and project outcomes (considered as project success and satisfaction with the project). We hypothesized that the PR’s skills would be positively associated with project success and satisfaction. This examination was based on a survey of a sample of PRs in public research institutions (mainly universities) in Italy and Poland. The choice of these two countries was motivated by the following: 

Both countries share the mission of becoming more innovative and more competitive in the European Union [31]. Both countries’ university research systems share many similarities, such as centralization, which means that public funds are distributed among universities on the basis of evaluation indicators, and non-compliance with standards can result in research funds being cut [32,33,34]. In both countries, the PR is a peer among peers, has no formal authority over the research team, is selected based on scientific results rather than managerial capabilities, and generally lacks formal knowledge of project management methodologies [35]. 

However, there are several differences that are likely to influence the attitudes and behaviors of PRs in both countries: 

Italian researchers have a longer history and more experience working with the present system or a similar one. Initial changes leading to the current system began in the 1980s, and the final shape of the evaluation system was achieved in 2004 [32,34]. On the other hand, it was only in 2004 that Poland entered the European Union. In the 1990s, Poland underwent deep transformations from a communist system, both politically and in the academic arena. This difference in time and experience may influence the mentality and attitudes of researchers in both countries. Furthermore, the governance of the Italian public research system is unstructured (where the system is understood to be the procedures and organizational and management tools designed to integrate and coordinate the generation, dissemination, and application of knowledge). Meanwhile, the problem that Poland must solve is how to adopt new measures that will enable it to keep pace with other countries. Italy presents a divided R&D system with a number of inefficient agencies, which have failed to build networks that would allow them to work together to achieve the required objectives. Though Poland has multiple research organizations that are well connected to each other, it has a high proportion of research that is not focused on specified social and economic goals [31]. For these reasons, a comparative study concerning project management in both countries could be of interest. 

Another aim was to propose directions of changes at public research institutions to help achieve greater project management maturity [36] and more efficient and satisfying use of public money.

Finally, we intended to indicate further research paths to study the interrelations between research project success, the satisfaction of project stakeholders, project management self-efficacy, team management self-efficacy, project-team collective efficacy, and empowering leadership.

## 2. Theoretical Background and Literature Review

As the objective of our paper was to examine the relationship between the PR’s skills on one hand and project outcomes on the other hand for projects implemented at aca-demic research centers, we conducted literature research concerning the PR and his or her skills, project success, and the relationships between the two aspects. Our literature research was thus divided into four parts: the success of research projects according to the existing literature; specific features of project teams in academic research projects; the role of soft skills in R&D project management and the relationship between leadership; and PR efficacy beliefs, and project success. The literature analysis was conducted using a systematic review method [37].

### 2.1. The Success of Research Projects According to the Existing Literature

Project success was defined in the literature in many different ways [38]. According to [30], project success criteria strongly depend on project type, so success criteria need to be explicitly defined for research projects. They can be found in numerous papers [39,40,41,42,43].

The proposed criteria are quantitative or qualitative. Quantitative criteria include (we present here a selection of criteria listed in [39,40,41,42,43]): the discounted cash flow generated by the project; the number of team members trained in project management due to the project’s realization; the probability of technological and commercial success of the project’s product; new scientists gained by the organization due to the project; the total income generated by the project; the number of patents and copyrights gained due to the project; the number of spin-offs started due to the project; the number of new projects funded due to the project; the number of papers published due to the project (possibly weighted by journal classification); the number of citations generated due to the project (possibly weighted by journal classification); the number of dissertations accomplished due to the project; the number of reports issued due to the project; the number of technology innovations worked out due to the project; the number of seminars organized cue to the project; the number of technology transfers resulting from the project; and positive publicity.

Qualitative criteria include: the organizational performance improvement achieved due to the project; customer satisfaction with the product of the project; the congruence with the strategy of the organization realizing the project; synergy with other projects realized by the organization; project team satisfaction, including project manager satisfaction; personal development of project team members; improvement of the organization’s reputation; new knowledge acquired due to the project; the technical gap size covered by the project product; and the newness of the technology used. 

Thus, project manager satisfaction is one of the success criteria for research projects. It refers to the PR’s attitude toward the project’s attained goals. In the literature, specific job satisfaction refers to an employee’s sense of success and achievement in a particular feature of their job. It is the key factor that leads individuals towards increased income, recognition, promotion, and the achievement of other objectives, resulting in a feeling of fulfillment [44] and improved workplace behavior and performance [45,46]. Finally, cooperation and communication care additional project success factors belong to project success factors [47,48,49,50].

### 2.2. Specific Features of Project Teams in Academic Research Projects

The existing literature indicates numerous important differences between academic and commercial research teams. Many of these differences imply that soft skills may be more important in academia than in commercial contexts [51,52,53]. Thus, academic and commercial teams differ concerning:

Composition [54]: Academic research teams typically consist of senior researchers and several junior researchers (masters and PhD students or post-docs), where some of the junior researchers do not work full-time for the university. In the commercial context, project teams are composed of scientists who have graduated from higher education institutions and work full-time in commercial organizations. Team members who do not work full-time in an organization and are not bound by a formal employment contract need more soft motivation than those employed formally. 

Basic objective: The aim of a commercial research team is to produce a profit or other tangible benefit for the organization (which includes the project team), and so team members have a common interest in striving for project success. University research teams, however, usually aim toward obtaining degrees and scientific titles, publishing papers in acknowledged scientific journals, and winning grants in calls for research projects, which are all individual-related objectives. The evaluation systems in most countries, including the two countries in this study (Italy and Poland), are based mainly on metrics relating to national databases of journals [55,56,57], which do not foster the pursuit of common goals. Therefore, university project team members need more informal motivation to work in the interests of the group. 

In [58], we read that research groups behave differently in academic and commercial settings. For example, the authors proved that commercial research team leaders’ affective exchanges with their group members were inversely related to the number of publications produced, whereas for academic teams the relation was exactly the opposite: Affective relations among the PR and the team increased the number of publications.

These arguments show that research project teams in universities need a PR who has sufficient soft skills, perhaps to a higher extent than commercial teams do.

As Hall [59] highlighted, the literature on teams features three main factors that can influence team effectiveness and, consequently, project success: cognitive, motivational, and behavioral features. Examples of such factors are: trust developed by team members, cooperation, and willingness to openly discuss problems and resolve conflicts linked to the division of responsibilities. Transfer among researchers positively affects team effectiveness and research [10,14,59,60]. Ernø–Kjølhede [10] stressed the importance of creating a sense of commitment and mutual obligation within the team as well as investing time and effort in confidence creation so that team members are willing to strive for common objectives and shared results. Cooperation should be set up in such a way as to avoid clashes between the individual needs of the team members and the project. Hall [59] highlighted that trust between team members can enhance knowledge-sharing and coordination. Studies carried out in organizations demonstrate that when members cooperate, they may share convictions and attitudes and thus show comparable persuasive and personal conduct standards [61,62]. In addition, group members must coordinate their work with others but are influenced by the beliefs, motivations, and performance quality of their collaborators [63]. From a socio-cognitive perspective, collective efficacy beliefs are leading indicators of a system’s functioning capacity [20]. Leadership qualities inspire greater collective efficacy levels, and teams demonstrating higher collective efficacy perform better in competitive situations, which suggests a positive relationship between collective efficacy and team performance [63,64].

### 2.3. The Role of Soft Skills in R&D Project Management

The PR can be defined as the leading scientist in publicly-funded academic research projects and programs [65], and it is the PR who is considered the research project manager in such contexts. Literature shows that the PR identity is still emerging and remains ill-defined, although initial attempts to define this role were made more than 10 years ago [65,66].

PRs in academic settings need project management knowledge and administration skills [67], but they do not necessarily possess them to a sufficient degree. This is due to many factors, including the specificity of academic organizations, the education of scientists, and the method of nominating PRs. At this level, appointing a PR is crucial, as it may be one of the main features that differentiates PRs from project managers in business. Scientists become PRs based on two groups of factors: push factors and pull factors [6,7]. Pull factors occur when the PR has a wish or desire to assume the role, and these including career ambition and personal drive. Push factors are project dependencies and institutional pressures, and these factors often determine a PR’s nomination. For example, scientists become PRs when they are assigned research funding in a call for projects. Project management knowledge and skills are rarely considered, although proposals to do so have been formulated [3,68]. The academic system measures achievements based on publications and other research factors rather than on management skills [3].

However, literature shows that soft competencies play an essential role in research project management [69,70,71,72,73,74,75,76], and PRs in the academic context need managerial skills [35,43,53]. In academic research management, leadership skills and self-efficacy are shown to be crucial: Leadership weaknesses are listed among the main problems in research project implementation and prevent projects from achieving success [43,53,77]. Among the soft skills, leadership has been shown to be one of the success factors for research projects [43,52,53].

Literature on leadership styles emphasizes effective knowledge management [78,79], as empowering leadership is considered consistent with the transition into a knowledge economy where individual autonomy is accentuated. Empowering leadership is positively correlated with knowledge sharing and team efficacy, which are both positively correlated with performance [80]. It has a strong influence on the creative and absorptive capacities of research teams [18,81] and plays a central role in shaping an organization’s ability to both exploit existing group capabilities and explore new technological domains [82].

An important practical and academic question is how to measure project managers’ skill levels [80]. From the social cognitive perspective, self-efficacy is the best predictor for future task performance [20]. This is particularly true when the task is challenging and requires a moderate-to-high level of effort [81]. In this context, the social cognitive model of leadership [82,83] claims that enhancing efficacy beliefs should be an important objective for improving leadership quality in organizations [84,85,86].

### 2.4. The Relationship between Leadership, PR Efficacy Beliefs, and Project Success

Literature studies highlight the relationship between project success and overall leadership in all sectors [87,88,89,90], including green building projects [91] and IT projects [92].

Research papers have found specific relationships between project success and transformational and empowering leadership in all sectors [93,94], including development and construction projects [95,96,97], as well as higher education projects [95]. The social cognitive model of leadership [83] has shown that project manager efficacy beliefs are related to project success [83].

In public research settings, researchers have reported that self-efficacy beliefs are correlated with other self-beliefs, motivation constructs, and carrier choices, changes, and achievement [98]. However, effect sizes and relationships depend heavily on how self-efficacy and criterial tasks are operationalized and assessed [99].

Our study includes two specific efficacy beliefs: (a) project management self-efficacy, which refers to how efficiently a PR manages resources and processes to achieve the organization’s operational goals; and (b) team management self-efficacy, which gauges how efficiently a project manager manages their team to achieve the project’s goals [83,84].

When employees are encouraged, they feel more competent after task completion [100]. It is not by accident that existing research highlights team leader behavior as one of the critical factors in successful team performance [101]. An empowering leadership style elicits the need for autonomy, emphasizing that employees should be engaged in matters that concern them and made part of and have a say in the larger whole [102].

PRs leading teams with high levels of collective efficacy could orient team behavior toward common planning. Collective efficacy motivates or demotivates the individual behavior of group members, influencing both the group’s goals and its commitment to achieving them [103,104]. Although empowering leadership has been recognized as essential to team performance for some time now [80], only a small number of papers have examined the mechanisms linking these variables. In our research, we included a measure of perceived team effectiveness (collective efficacy), with the intention of analyzing PR perceptions of how teams dealt with the different project objectives.

In research projects, these skills may be critical determinants of successful outcomes and satisfaction with the project’s realization [105,106].

## 3. Materials and Methods

A quantitative study was designed as the most appropriate research method for our aims, in line with the previous studies on the subject [1].

### 3.1. Participants and Procedure

The study was conducted in Italy and Poland. The research was performed within three weeks (September 2019). All participants were informed about the nature of the study and assured that their responses would remain anonymous. All respondents provided consent to be included in the study and participated voluntarily. They did not receive any compensation for their participation.

We aimed to reach managers of complex research projects (not programs or portfolios) implemented on the level of a single research institution (thus, not coordinators of multinational and multinational projects). The term “complex project’ is understood here as proposed by Kerzner [107]. The author did not propose a strict definition but stated that features that may decide about project complexity are: size, dollar value, uncertainty, complex interactions, etc. In addition, in other models (e.g., [108] project complexity is measured using budget and duration. The majority of research projects implemented at public universities have a duration of about 2–3 years. All of them are linked to uncertainty, simply by being research projects. Thus, we decided to consider projects with budgets over €100,000.

In Italy, participants were selected from researchers at the University of Naples Federico II, a public university, one of the largest universities in Southern Italy. The participants should have managed at least one complex research project (defined as above, there were PRIN, FIRB, or Horizon 2020 projects), and their projects should have ended within the past 2 years.

The PRIN is a fund of the Italian research ministry which finances projects on various research topics. The funded projects are coordinated by a scientific manager, who reports to an Italian university. The implementation of the project involves a number, generally high, of research groups coordinated by managers belonging to other national universities. The FIRB is a fund that finances research projects presented by researchers under the age of 40. Financeable projects include basic research projects wherein a high scientific and technological content are funded, relating for example, to the development of pervasive and multisectoral technologies or the enhancement of large public or public-private research infrastructures.

These projects can be defined as complex, in the light, for example, of the criteria relating to complexity proposed by Vidal and Marle [108]: size (duration, number of activities, number of professional resources involved); degree of diversification and geographical location of stakeholders and diversification of professional skills; interdependencies between activities, sharing of information, interactions between stakeholders; environmental context (differentiation of the economic and socio-cultural contexts of the Italian regions). In February 2019, there were 48 projects with these characteristics at the University of Naples Federico II. Participants were all invited to fill out an online questionnaire on the SurveyMonkey platform. Local university data and personal contacts were used to identify them. Twenty-eight PRs agreed to participate in the study. Their average age was 57.25 years (*SD* = 9.61, range = 36–71 years). With regard to academic roles, 64.3% of the PRs were full professors, 14.3% associate professors, 3.6% researchers without professor positions, 10.7% temporary staff, and 7.2% other research staff. The recruitment was performed by asking the project office of the University of Naples Federico II for a list of PR of projects with the characteristics just described. Then we used an open email from one of the co-authors of the paper, explaining the context and the objective of the study.

In Poland, the participants were recruited by a professional research firm, which used the CATI survey procedure [109]. There were two inclusion criteria. First of all, the participants were recruited from public institutions that dealt with scientific R&D projects, such as public universities, public R&D companies, or the R&D departments of public administration. Secondly, the participants should have had at least 5 years of professional experience in managing scientific complex research projects and research teams. This more severe criterion regarding the length of professional experience was chosen for the Polish sample because the members of this sample were completely unknown to the authors and their expertise could not be controlled informally, as was the case for the Italian sample. The recruitment was performed entirely by the external research company. They used an open letter from one of the co-authors of the paper, explaining the context and the objective of the study.

The original survey pool was made up of 45 participants. After accounting for the inclusion criteria, 6 participants were kept out of the study due to a short professional experience in managing R&D projects. Thus, in the Polish study, the data were obtained from 39 participants. The participants’ average age was 40.9 years (*SD* = 10.4, ranging from 25 to 67 years). Twenty percent of the participants were full professors, 46.2% associate professors, 10.3% did not hold professor positions, 12.8% were temporary researchers, and 10.3% were other research staff.

The difference in participant selection procedures in Italy and Poland was caused by difficulties in getting access to appropriate candidates. The set of appropriate candidates was per se small and numerous potential participants refused to participate because of lack of time. Thus, in Italy participants from the home university of the Italian co-authors were asked to participate, which increased their motivation. The Polish co-authors were unable to find a sufficient number of respondents; thus, they asked a professional company, who motivated the interviewees using their own methods.

In total, 67 PRs participated in this study. The detailed characteristics of respondents are shown in Table 1.

### 3.2. Measures and Analysis

The selection of measures was carried out using literature analysis of the variables under study and their measurement in the field of project management. We took into account: (a) anchorage to the literature of interest, the scope of specific functioning (especially in the case of self-efficacy, see Bandura, 1997); (b) instruments already validated in previous studies and their reliability; the agility of administration (instruments with less than 15 items were preferred, when possible).

All measurement instruments were validated in the Italian language and then translated from Italian to Polish by a management expert with experience in translating survey questionnaires. The study questionnaires were then back-translated into the original language and checked by independent translators in terms of language accuracy and equivalence of both versions, i.e., Italian and Polish. Upon mutual agreement between the authors, some changes have been introduced in the original scales used in the study. In particular, some items of the Empowering Leadership Scale and Project Management Self-Efficacy Scale were adapted to the context of academic research, by changing, for example, words such as project group, into research group.

#### 3.2.1. Project Success

To measure the project success we chose, among the success criteria proposed by the literature, 7 items expressing different aspects of project success: (1) new knowledge development, (2) members knowledge integration, (3) number of publications, (4) patents development, (5) spin-offs development, (6) department reputation growth, and (7) facilitating the acquisition of new sources of funding [110]. In this study, we used a total score as the project success index, assuming equal weights. Cronbach’s alpha for the total score was 0.70 in the Italian group and 0.92 in the Polish group.

#### 3.2.2. Satisfaction with the Project

Satisfaction with the project was assessed by 1 item [111]: “I am satisfied with the entire course of the project”. Respondents responded on a 7-point scale, from 1 “disagree entirely” to 7 “agree entirely”.

#### 3.2.3. Empowering Leadership

Empowering leadership was measured with the Empowering Leadership Questionnaire (ELQ) [112]. The ELQ consists of 38 items diagnosing 5 leadership dimensions:

(1) Leading by example (5 items) refers to a set of behaviors that show the leader’s commitment to his or her work as well as the work of his or her team members, including behaviors such as “working harder than other team members”. A sample item: “Works as hard as anyone in my work group.” In our study, 3 items were used Cronbach’s alpha in the Italian sample was 0.68, and in the Polish sample 0.60;

(2) Participative decision-making (6 items) refers to acknowledging team members’ knowledge and expert contribution in making decisions. A sample item: “Encourages work group members to express ideas/suggestions.” The internal consistency of this subscale was satisfactory (Italian group: α = 0.73, Polish group: α = 0.75);

(3) Coaching (11 items) refers to a set of behaviors that educates team members and helps them to become self-reliant. A sample item: “Suggests ways to improve my work group’s performance”. Cronbach’s alpha was 0.84 in the Italian group, and 0.86 in the Polish group;

(4) Informing (5 items) refers to the leader’s dissemination of project-wide information, such as mission and philosophy, as well as other important information. A sample item: “Explains rules and expectations to my work group”. The reliability of this subscale was high (Italian group: α = 0.86, Polish group: α = 0.79);

(5) Showing concern (10 items) expresses a collection of behaviors that demonstrates a general regard for team members’ well-being. A sample item “Shows concern for work group members’ well-being”. Cronbach’s coefficient for the Italian group was 0.83, and for the Polish group.83. Respondents provided answers on a 5-point scale (1 “never” to 5 “always”).

#### 3.2.4. Project Management Self-Efficacy

To measure project management self-efficacy, a short version of the Project Management Self-Efficacy Scale (PMSE-S6) [83] was used. This questionnaire includes 6 items measuring how efficiently PR manages resources and processes to achieve the organization’s operational goals. A sample item: “I evaluate project reviews and suggested improvements, discuss with key stakeholders, and take appropriate action”. A 5-point Likert-type scale was used to capture the extent of agreement, ranging from 1 “I cannot do the task (0% confident)” to 5 “totally confident to manage the task effectively (100% confident)”. Cronbach’s alpha for the entire scale was 0.92 in the Italian group and 0.86 in the Polish group.

#### 3.2.5. Team Management Self-Efficacy

To measure self-efficacy in team management, a composite measure of 10 items, gauging how efficiently the project manager manages the tam to achieve the project’s operational goals [110] was used. A sample item: “I can encourage members of the working group to be involved and interested in the project”. A 5-point Likert-type scale was used to capture the extent of agreement, ranging from 1 “I cannot do the task” to 5 “I am totally confident to manage the task effectively”. Cronbach’s alpha for the entire scale was 0.94 in the Italian sample, and 0.90 in the Polish sample.

#### 3.2.6. Team Collective Efficacy

Team collective efficacy in the project was assessed with 5 items measuring how the team dealt with the different objectives of the project [110]. An example item is, “Each team member accepted their role in the project, and there were no problems with the division of tasks”. Responses were given on a 5-point Likert scale (from 1 “completely disagree” to 5 “completely agree”). Cronbach’s alfa coefficient was 0.83 for the Italian group and 0.94 for the Polish group.

Survey data were then entered into the SPSS 26.0 database and verified by project staff for accuracy. To compare Italian and Polish research groups regarding PR management skills and project success indicators, a one-way analysis of variance (ANOVA) was conducted. Relationships between the analyzed variables were examined by Spearman’s correlation analysis, which was conducted for the total Italian–Polish sample.

## 4. Results

### 4.1. Differences in the Measured Variables between Italian and Polish PR’ Groups

In Table 2 descriptive statistics for measured variables in Italian and Polish project managers are presented.

The ANOVA analysis results showed a significantly higher mean value for team management self-efficacy (*M* = 4.18 and *M* = 3.88 respectively) and leading by example (*M* = 4.35 and *M* = 4.07 respectively) in the Polish sample in comparison to the Italian sample. Next, the ANOVA results indicated significant differences in the mean values of satisfaction with the project between the Italian and Polish dataset. There were no significant differences between the Italian and Polish PRs in terms of project success, or project management self-efficacy, and team collective efficacy. Assessment of empowering leadership in the project (in terms of subdimensions and aggregated scores) were not significantly different between the Italian and Polish PRs groups, either.

Due to a small number of differences in project management scores between Polish and Italian PRs, both groups will be considered jointly in further analyses.

### 4.2. Relationships between the Measured Variables

As depicted in Table 3, all correlation coefficients were statistically significant and positive.

As depicted in Table 3, all correlation coefficients were statistically significant and positive. Two project outcomes, i.e., project success and satisfaction with the project, were intercorrelated at a moderate level. Project success was also moderately correlated with PR’s skills of empowering leadership, team and project management self-efficacy, and team collective efficacy. Satisfaction with the project, as the second project outcome, was correlated at a moderate level with all PR’s skills of empowering leadership, team and project management self-efficacy, and team collective efficacy. All variables representing PR’s skills were intercorrelated at a moderate and high level.

## 5. Discussion and Conclusions

As Huljenic et al. [2] highlighted, research projects in industry differ significantly from pure academic research projects in how they are planned and managed.

In the academic context, contrary to what happens in industry, PRs often lack knowledge and skills related to project management systems, formal authority over the project team researchers (they are “peers among peers”), and administrative project support [3,4].

The research presented in this paper investigated a relatively understudied topic: the relationship between PR management skills (such as leadership style and self-efficacy beliefs) and the success of research projects, including satisfaction with the project. The study took into consideration cross-cultural differences and was conducted in Italy and Poland in public research institutions. The subject is of the highest importance not only for the two countries represented in this study but in fact for every country in the world: each government spends a considerable amount of money on public research projects, and it is crucial to guarantee their high success rate.

The results showed a significantly higher mean value for team management self-efficacy in Polish PRs compared to Italian PRs. This is probably due to the greater experience in research coordination declared by Polish PRs. In Poland, eligible study participants had at least 5 years’ professional experience managing scientific research projects and research teams. In Italy, they should have at least followed a complex project in the last 2 years. Managing a project team is quite different from managing other types of groups, thus having experience in this area promotes self-efficacy. This is congruent with Bandura’s assertion that the most influential source of self-efficacy is performance accomplishments [20].

The results also show that Italian PRs reported higher project satisfaction ratings than Polish PRs. This may be due to various reasons: the research challenges faced in Italy, integrating and coordinating various innovation processes in order to secure its place among the most innovative countries in Europe; as well as, a longer experience of implementing research projects in Italy than in Poland [31].

As highlighted in the general correlation matrix, the soft skills considered in our study were related to the selected criteria of project success and satisfaction with the project, in line with the hypotheses stated in the Introduction (that the PR’s skills would be positively associated with project success and satisfaction).

In the public research context, Ricketts and Bruce [113] suggested that leaders need to continually encourage research teams. The probability of success in public research projects could be increased by balancing management weaknesses with an adequate value of team efficacy. In such cases, team efficacy would be based on trust and cooperation between team members, willingness to transfer knowledge, and willingness to openly discuss problems and resolve conflicts. Our results are coherent with these statements: they highlighted a significant correlation between empowering leadership and a team’s collective efficacy as perceived by the PR. Collective efficacy belief stems from the effects of mastery and vicarious learning experiences, group cohesion, and the emotional tone of the group [114]. In empowering leadership, leaders delegate authority to their employees and increase motivation by giving them more responsibility and autonomy in their work. Empowering leadership might contribute to collective efficacy (and team effectiveness) through each of these mechanisms [115].

In (complex) research projects in public research centers, the success of the project is linked not only to the leadership of the project manager (as we mentioned in the literature review) but also to other variables, which constitute a peculiarity of the management of research projects. The scarce formal recognition given to the PR in their role as project manager and their limited project management knowledge and skills are critical elements for project success in public research. On the other hand, self-efficacy does not represent a generalized feeling of control, but rather an individual’s comprehensive judgment of their capability to perform a particular job [116]. As proposed by the social cognitive theory [20] and supported by empirical findings [83], efficacy beliefs can predict future performance. Thus, strengthening the specific self-efficacy of the PR could be a way to influence the whole project process, favoring the PR functioning as research project manager.

We also found that empowering leadership is significantly related to the team manager’s self-efficacy. Indeed, the team manager’s self-efficacy refers to how efficiently the project manager handles the team to achieve project goals. To promote empowering leadership, organizations should be interested in reinforcing managers’ efficacy beliefs. Our study suggests that empowering leadership and efficacy beliefs can affect project satisfaction. From a management perspective, in order to attain good performance, it is essential to match leadership style not only with efficacy beliefs but also with worker satisfaction and well-being. With its emphasis on employee autonomy, motivation, and development, the logic of empowering leadership appears to be well suited to a knowledge-based employment approach [117].

To sum up the above discussion, we can claim that it is of utmost importance for public research institutions to promote team efficacy, PR’s self-efficacy, and empowering leadership. This line of action will increase the probability of research projects’ success, including satisfaction with the projects. This will be beneficial to all the parties involved: the organizations as a whole, their members, and the national economies and societies. In the next sections, we translate this postulate into specific proposals.

### 5.1. Future Research Directions

The results of this study suggest that project leadership and soft skills are fertile ground for further research. It would be useful for future studies to investigate factors that facilitate project success, satisfaction with the project, and empowering leadership styles. These factors should be identified based on the literature on research project success and management (for example, an important potential factor is the organizational project management maturity), but also through interviews or surveys, so that factors that have not been identified in the literature but are important can be considered.

**Figure 1:**
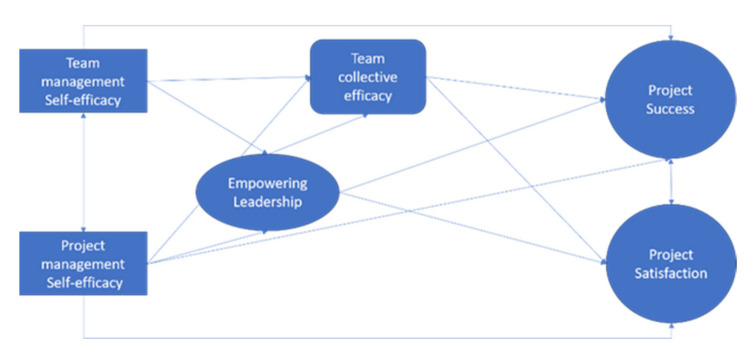
Future research paths pattern map: testable hypothesized model.

Based on our findings and literature analysis, we created a pattern map of suggested future research paths (Figure 1). These represent testable hypotheses that can be pursued in empirical studies.

**Proposition** **1.** *Project success and project satisfaction are outcome variables [118,119,120,121].*

**Proposition** **2.** *Team management self-efficacy and project management self-efficacy affect empowering leadership, collective efficacy, project success, and satisfaction with project performance [20,80].*

**Proposition** **3.** *Empowering leadership affects collective team efficacy, and both affect project satisfaction and project success [1,122,123].*

### 5.2. Limitations

A number of limitations of the present study should be highlighted. Firstly, due to the cross-sectional nature of the data, the results were based on correlational analysis, and causality could not be inferred. Inclusion criteria for our study participants were quite precise, which resulted in a small sample size (N = 67). This is indeed a limitation that needs to be addressed in future studies, mainly for the reasons of building empirical models which would explain more complex interrelationships.

The estimated correlations (and their directions) indicate the need for further research to identify the institutional and personal determinants that may underlie the pathways for these associations. Longitudinal studies should tap into the causal nature and stability of the investigated relationships. The self-reported data are not immune to common method biases, among others the multicollinearity. Therefore, future studies need to assess these variables, using independent sources of information to reduce the method variance and offer additional confidence in hypotheses. The two studies used different questionnaire administration methods (CATI vs. Web-Based Survey Responses). However, in line with literature that states how differences in two methods occur, especially when considering sensitive research topics (such as sexting and body image) and groups that are being recruited (young people, males, and NEET [124]), the results should not be affected by the different methods across the two countries.

Moreover, self-report instruments have the potential for social desirability bias. Although we need to consider this limitation, it is reasonable to think that this bias does not significantly influence our data, as anonymity was guaranteed in the data’s collection [125]. In addition, the Leading by Example Scale yielded a low internal consistency both in Italian and Polish participants (0.68 and 0.60, respectively). However, given that all other scales had acceptable alphas, the value was not considered a threat to the validity of the study results [126].

Finally, the socio-demographic differences and the different levels of project management experience between the two samples could obscure any firm comparisons and conclusions. This shortcoming may be mitigated by enlarging both samples in future studies.

Our study provides preliminary data on significant differences between the two sample groups in relation to leadership style and success criteria. However, we are cautious about making far-reaching cultural inferences, as more various design studies are needed to be able to compare the two professional groups. Team perception of leadership and teamwork is a vantage point for assessing existing research and is associated with quality of team interaction [115]. In future studies, it would be essential to analyze the relationship between a PR’s soft skills and those of the other research team members. In addition, as we know that gender norms [127] can shape satisfaction levels, it would be important to address also this issue.

Another important limitation is the assumed definition of research project success. As stated in the literature review, measuring this success is a complex issue. For our study, we chose a selection of possible research project criteria and gave them all the same weight. Additionally, we distinguished one criterion: the satisfaction of the PR with the whole project course. In future research, other approaches to measuring research project success should be considered.

Despite the above limitations, we are persuaded that this paper delivers an important contribution to the problem of how to spend public money on research in an efficient way. It indicates concrete steps to include in public research institutions strategies already in the present moment and opens new research paths which are bound to provide new insights for public research institutions management.

### 5.3. Practical Implications

Even if this study’s cross-sectional nature makes it difficult to determine causality, our findings may have implications for interventions targeted at PRs in public research centers. Planning development programs and their evaluation methods in a more targeted and effective way means considering factors such as efficacy beliefs and leadership style to improve project satisfaction and success. The popularity of leadership development programs indicates that leadership effectiveness is of primary concern to organizations [128]. The nature of project management in academia has changed substantially in the last few years, becoming more complex and cognitively demanding [129]. Highly skilled and educated PRs could become the core of the rapidly growing public research segment. Yazici [130] found that organizations that have more stakeholder-participation, cohesion, and shared values and commitments are more likely to achieve project success. However, this can only occur when individuals with appropriate psychological skills are present in the organization [131]. In light of such organizations’ peculiarities, managers of public research centers, understanding the role of these factors, could identify levels of intervention to establish more mature project cultures. They could utilize PR project management skills to achieve higher levels of research productivity. This is a priority of both the Italian and the Polish governments, as the two countries share the mission of becoming more innovative and competitive. Furthermore, both countries need to develop good practices and innovative solutions to support R&D activities and increase research productivity.

The interventions to be considered in public research centers could be of various types, e.g.:

- Improved project management training for PRs, with attention to the development of empowering leadership;
- Increased focus on developing soft skills among research team members to develop a propensity for communication, cooperation, and motivation;
- Work on implementing reward systems that consider the scientific production of researchers and the evaluation of behaviors such as interoperability, cooperation, and group work.

Finally, one of the ways research organizations could improve efficiency and performance is to empower their employees and let them share their knowledge and experiences, so that they may learn from each other.

## Figures and Tables

**Table 1:** Characteristics of respondents (N = 67).

	Italy	Poland
Participants N	28	39
Women	5	18
Men	23	21
Age range (in years)	36–71	25–67
Age M (SD)	57.3 (9.61)	40.9 (10.4)
Full professor	17 (64.3%)	8 (20.5%)
Associate professor	4 (14.3%)	18 (46.2%)
Researchers without professor position	1 (3.6%)	5 (12.8%)
Temporary staff	3 (10.7%)	5 (12.8%)
Other research staff	2 (7.2%)	4 (10.3%)

**Table 2:** Descriptive statistics for measured variables in Italian and Polish samples.

	Italian PRs (*n* = 28)			Polish PRs (*n* = 39)			*F* (1, 65)	*Eta* ^2^
	*M*	*SD*	*SE*	*M*	*SD*	*SE*		
1. Project success (mean score)	4.08	0.64	0.12	4.03	0.92	0.15	0.063	0.001
2. Satisfaction with the project	6.32	0.48	0.09	5.62	1.73	0.28	4.425 *	0.064
3. Empowering leadership (mean score)	4.11	0.54	0.10	4.13	0.49	0.08	0.019	0.001
3.1. Leading by example	4.07	0.69	0.13	4.35	0.55	0.09	3.364	0.049
3.2. Participative decision making	4.07	0.37	0.07	4.01	0.52	0.08	0.247	0.004
3.3. Coaching	4.13	0.53	0.10	4.18	0.54	0.09	0.147	0.002
3.4. Informing	4.36	1.71	0.32	4.14	0.61	0.10	0.578	0.009
3.5. Showing concern	3.88	0.58	0.11	4.10	0.53	0.08	2.704	0.040
4. Project management self-efficacy	3.96	0.71	0.13	4.19	0.66	0.10	1.833	0.027
5. Team management self-efficacy	3.88	0.64	0.12	4.18	0.53	0.08	4.582 *	0.066
6. Team collective efficacy	3.91	0.68	0.13	3.95	1.04	0.17	0.043	0.001

*Note*. * *p* < 0.05.

**Table 3:** Correlation matrix for all measured variables (Spearman correlation coefficients).

	*M*	*SD*	1	2	3	4	5	6
1. Project success (mean score)	4.06	0.81	1					
2. Satisfaction with project	5.91	1.39	0.46 **	1				
3. Empowering leadership (mean score)	3.79	0.47	0.47 **	0.37 **	1			
4. Project management self-efficacy	4.10	0.68	0.44 **	0.35 **	0.54 **	1		
5. Team management self-efficacy	4.06	0.59	0.38 **	0.34 **	0.71 **	0.65 **	1	
6. Team collective efficacy	3.93	0.90	0.37 **	0.44 **	0.49 **	0.50 **	0.51 **	1

*Note. N =* 67 ** *p* < 0.01.

## Data Availability

The data presented in this study are available on request from the corresponding author.

## References

[1] Geoghegan L., Dulewicz V. (2008). Do project managers’ leadership competencies contribute to project success?. Proj. Manag. J..

[2] Huljenic D., Desic S., Matijasevic M. Project management in research projects. Proceedings of the International Conference on Telecommunications.

[3] Cassanelli A.N., Sánchez G., Guiridlian C. (2016). Principal researcher vs. R&D Project manager: Who should drive R&D?. R&D Manag..

[4] Divjak B., Kukec S.K. (2008). Teaching methods for international R&D project management. IJPM.

[5] Riol H., Thuillier D. (2016). Project management for academic research projects: Balancing structure and flexibility. IJPOM.

[6] Fowler N., Lindahl M., Sköld D. (2015). The projectification of university research: A study of resistance and accommodation of project management tools & techniques. Int. J. Proj. Manag..

[7] Cassanelli A.N., Cantú A., Moreno J., Rossetti G., Arcusin L., De Greef M. (2017). Instrument design to diagnose R&D project management activities at universities. IJOPM.

[8] Kisielnicki J. (2014). Project management in research and development. Found. Manag..

[9] Jordan G., Hage J., Mote J., Hepler B. (2005). Investigating differences among research projects and implications for managers. R&D Manag..

[10] Ernø-Kjølhede E. (2000). Project management theory and the management of research projects. MPP.

[11] Kuchta D., Skowron D. (2016). Classification of R&D projects and selection of R&D project management concept. R&D Manag..

[12] Ungureanu A., Ungureanu A. (2014). Methodologies Used in Project Management. Ann. Spiru Haret Univ..

[13] Kuchta D., Skorupka D., Duchaczek A., Kowacka M. Modified, stakeholder’s perspective-based DEA approach in IT and R&D project ranking. Proceedings of the ICEIS.

[14] Vansteenkiste M., Sierens E., Goossens L., Soenens B., Dochy F., Mouratidis A., Aelterman N., Haerens L., Beyers W. (2012). Identifying configurations of perceived teacher autonomy support and structure: Associations with self-regulated learning, motivation and problem behavior. Learn. Instr..

[15] Vom Brocke J., Lippe S. (2015). Managing collaborative research projects: A synthesis of project management literature and directives for future research. IJOPM.

[16] Dewulf A., Bouwen R. “Doing Differences”: The Emergence of Differences in Issue Framing in Multi-Actor Conversations. Proceedings of the IACM.

[17] Sharma P.N., Kirkman B.L. (2015). Leveraging leaders: A literature review and future lines of inquiry for empowering leadership research. Group Organ. Manag..

[18] Zhang X., Bartol K.M. (2010). Linking empowering leadership and employee creativity: The influence of psychological empowerment, intrinsic motivation, and creative process engagement. Acad. Manage. J..

[19] Kirkman B.L., Rosen B. (1997). A model of work team empowerment. ROCD.

[20] Bandura A. (1997). Self-Efficacy: The Exercise of Control.

[21] Capone V., Petrillo G. (2016). Teachers’ perceptions of fairness, well-being and burnout: A contribution to the validation of the Organizational Justice Index by Hoy and Tarter. Int. J. Educ. Manag..

[22] Pajares F. (1996). Self-efficacy beliefs in academic settings. Rev. Educ. Res..

[23] Marino L., Capone V. (2021). The role of personal resources to contrast the working students’ malaise: The mediating role of Work-Study Conflict in the relationship between self-efficacy and performance. Psicol. Della Salut..

[24] Argentero P., Cortese C.G. (2018). Psicologia Delle Organizzazioni.

[25] Cooke F.L., Wood G., Horwitz F. (2015). Multinational firms from emerging economies in Africa: Implications for research and practice in human resource management. Int. J. Hum. Resour. Manag..

[26] Kozlowski S.W., Ilgen D.R. (2006). Enhancing the effectiveness of work groups and teams. Psychol. Sci. Public Interest.

[27] Schein E.H. (2016). Organizational Culture and Leadership.

[28] Toor S.R., Ogunlana S.O. (2010). Beyond the ‘iron triangle’: Stakeholder perception of key performance indicators (KPIs) for large-scale public sector development projects. IJPM.

[29] Wang X., Huang J. (2006). The relationships between key stakeholders’ project performance and project success: Perceptions of Chinese construction supervising engineers. IJPM.

[30] Shenhar A.J., Dvir D., Levy O., Maltz A.C. (2001). Project success: A multidimensional strategic concept. LRP.

[31] Fino V., Rosiek J. (2016). R&D: Italy and Poland compared. Argum. Oecon. Crac..

[32] Baldissera A. (2004). La valutazione nelle università italiane: Processi organizzativi ed indicatori. Quad. Sociol..

[33] Lehmann E.E., Meoli M., Paleari S., Stockinger A.E. (2016). Approaching Effects of the Economic Crisis on University Efficiency—A Comparative Study of Germany and Italy. SSRN.

[34] Stachowiak-Kudła M. (2012). Autonomia Szkół Wyższych a Instytucjonalne Mechanizmy Zapewnienia Jakości w Polsce i Wybranych Krajach Europejskich.

[35] Kuchta D., Gładysz B., Skowron D., Betta J. (2017). R&D projects in the science sector. R&D Manag..

[36] De Souza T.F., Gomes C.F.S. (2015). Assessment of maturity in project management: A bibliometric study of main models. Proc. Comput. Sci..

[37] Kitchenham B., Charters S. (2007). Guidelines for performing Systematic Literature reviews in Software Engineering Version 2.3. EBSE.

[38] Martens C.D.P., Machado F.J., Martens M.L., de Oliveira e Silva F.Q.P., de Freitas H.M.R. (2018). Linking entrepreneurial orientation to project success. IJPM.

[39] Eilat H., Golany B., Shtub A. (2008). R&D project evaluation: An integrated DEA and balanced scorecard approach. Omega.

[40] Yuan B., Huang J.N., Thore S.A. (2002). Applying data envelopment analysis to evaluate the efficiency of R&D projects—A case study of R&D in energy technology. Technology Commercialization.

[41] Revilla E., Sarkis J., Modrego Rico A. (2003). Evaluating performance of public–private research collaborations: A DEA analysis. J. Oper. Res. Soc..

[42] Despotis D.K., Koronakos G., Sotiros D. (2015). A Multi-objective programming approach to network DEA with an application to the assessment of the academic research activity. Proc. Comput. Sci..

[43] Klaus-Rosińska A. Success of Research and Research and Development Projects in the Science Sector.

[44] Kaliski B.S. (2007). Encyclopedia of Business and Finance.

[45] Armstrong M. (2015). Armstrong’s Handbook of Performance Management: An Evidence-Based Guide to Delivering High Per-Formance.

[46] Capone V., Petrillo G. (2015). Organizational efficacy, job satisfaction and well-being: The Italian adaptation and validation of Bohn organizational efficacy scale. J. Manag. Dev..

[47] Liu W.H., Cross J.A. (2016). A comprehensive model of project team technical performance. IJPM.

[48] Bond-Barnard T.J., Fletcher L., Steyn H. (2018). Linking trust and collaboration in project teams to project management success. IJMPB.

[49] Baccarini D. (1999). The logical framework method for defining project success. Proj. Manag. J..

[50] Scott-Young C., Samson D. (2008). Project success and project team management: Evidence from capital projects in the process industries. J. Oper. Manag..

[51] Zhang Q., Yang S., Lei R. Enablers analysis of knowledge-sharing in university research innovation teams. Proceedings of the IMMS.

[52] Bartlett A.G., Kanowski P.J., Van Kerkhoff L., Byron R.N. (2017). Identifying factors that influence the success of forestry research projects implemented in developing countries: Case study results from Vietnam. J. For. Res..

[53] Kuchta D., Betta J., Jastrzębska J., Frączkowski K., Gładysz B., Prałat E., Ptaszyńska E., Rola P., Walecka-Jankowska K., Ropuszyńska-Surma E. Success and failure factors of R&D projects at universities in Poland and France. Proceedings of the 1st International Conference on Business Risk in Changing Dynamics of Global Village.

[54] Tkachenko O., Ardichvili A. (2020). Critical factors impacting interdisciplinary university research teams of small size: A multiple-case study. Team Perform. Manag..

[55] Ancaiani A., Fantoni S. (2014). La valutazione dei prodotti della ricerca ed il suo impatto sul sistema universitario e degli enti di ricerca: Il caso italiano. Melanges Casa Velazquez.

[56] Kulczycki A. (2017). Assessing publications through a bibliometric indicator: The case of comprehensive evaluation of scientific units in Poland. Res. Eval..

[57] Cohen N., Ochsner N.K. (2018). From surviving to thriving in the face of threats: The emerging science of emotion regulation training. Curr. Opin. Behav. Sci..

[58] Olsson L., Hemlin S., Pousette H. (2012). A multi-level analysis of leader-member exchange and creative performance in research groups. Leadersh. Q..

[59] Hall K.L., Vogel A.L., Huang G.C., Serrano K.J., Rice E.L., Tsakraklides S.P., Fiore S.M. (2018). The science of team science: A review of the empirical evidence and research gaps on collaboration in science. Am. Psychol..

[60] Barnes T., Pashby I., Gibbons A. (2002). Effective university–industry interaction: A multi-case evaluation of collaborative R&D projects. Eur. Manag. J..

[61] George J.M. (1990). Personality, affect, and behavior in groups. J. Appl. Psychol..

[62] George J.M., Jones G.R. (1996). The experience of work and turnover intentions: Interactive effects of value attainment, job satisfaction, and positive mood. J. Appl. Psychol..

[63] Capone V., Caso D., Donizzetti A.R., Procentese F. (2020). University student mental well-being during COVID-19 outbreak: What are the relationships between information seeking, perceived risk and personal resources related to the academic context?. Sustainability.

[64] Salanova M., Ortega-Maldonado A., Van Zyl L., Rothmann S. (2019). Psychological capital development in organizations: An integrative review of evidence-based intervention programs. Positive Psychological Intervention Design and Protocols for Multi-Cultural Contexts.

[65] O’Kane C., Mangematin V., Zhang J.A., Cunningham J.A. (2020). How university-based principal investigators shape a hybrid role identity. Technol. Forecast. Soc. Chang..

[66] O’Reilly P.T., Cunningham J.C. A model for studying the effectiveness of principal investigators in publicly funded research. Proceedings of the IEEE International Conference on Management of Innovation & Technology.

[67] Cunningham J.A., Mangematin V., O’Kane C., O’Reilly P. (2016). At the frontiers of scientific advancement: The factors that influence scientists to become or choose to become publicly funded principal investigators. J. Tech. Tran..

[68] Capaldo G., Iandoli L., Zollo G. (2006). A situationalist perspective to competency management. Hum. Resour. Manag..

[69] Ballesteros-Sánchez L., Ortiz-Marcos I., Rodríguez-Rivero R., Juan-Ruiz J. (2017). Project management training: An integrative approach for strengthening the soft skills of engineering students. Int. J. Eng. Ed..

[70] Carvalho M., Junior R. (2015). Impact of risk management on project performance: The importance of soft skills. Int. J. Prod. Res..

[71] Lent B., Pinkowska M. (2012). Soft skills needed in the ICT project management-classification and maturity level assessment. Int. J. Appl. Syst. Stud..

[72] Magano J., Silva C., Figueiredo C., Vitória A., Nogueira T., Dinis M.A.P. (2020). Generation Z: Fitting project management soft skills competencies—A mixed-method approach. Educ. Sci..

[73] Pinkowska M., Lent B. Soft skills needed in the ICT project management-scientists and practitioners’ awareness. Proceedings of the 15th IBIMA Conference.

[74] Rico D.F., Liebowitz J. (2014). Managing projects as though people mattered: Using soft skills and project management tools for successful enterprise transformation. Bursting the Big Data Bubble: The Case for Intuition-Based Decision Making.

[75] Wanivenhaus H., Kovač J., Žnidaršič A., Vrečko I. (2018). Vienna construction projects: Redirection of project management critical success factors—more focus on stakeholders and soft skills development. Lex Loc..

[76] Zuo J., Zhao X., Nguyen Q.B.M., Ma T., Gao S. (2018). Soft skills of construction project management professionals and project success factors: A structural equation model. Eng. Constr. Archit. Manag..

[77] Gładysz B., Kuchta D. Stakeholder Communication Impact on the Success of IT Project—Fuzzy Approach. Intelligent and Fuzzy Techniques in Big Data Analytics and Decision Making. Proceedings of the INFUS Conference.

[78] Oliver S., Kandadi K.R. (2006). How to develop knowledge culture in organizations? A multiple case study of large distributed organizations. J. Knowl. Manag..

[79] Singh S.K. (2008). Role of leadership in knowledge management: A study. J. Knowl. Manag..

[80] Srivastava A., Bartol K.M., Locke E.A. (2006). Empowering leadership in management teams: Effects on knowledge sharing, efficacy, and performance. Acad. Manage. J..

[81] Cavazotte F.S.C.N., Paula F.O. (2020). Too much of a good thing: The quadratic effect of shared leadership on creativity and absorptive capacity in R&D teams. Eur. J. Innov. Manag..

[82] Tushman M.L. (2017). Innovation Streams and Executive Leadership: R&D leadership plays a central role in shaping a firm’s ability to both exploit existing capabilities and explore new technological domains. Res. Technol. Manag..

[83] Blomquist T., Farashah A.D., Thomas J. (2016). Project management self-efficacy as a predictor of project performance: Constructing and validating a domain-specific scale. IJPM.

[84] Locke E.A., Latham G.P. (1984). Goal Setting: A Motivational Technique That Works!.

[85] McCormick M.J. (2001). Self-efficacy and leadership effectiveness: Applying social cognitive theory to leadership. J. Leadersh. Organ. Stud..

[86] Yang L.R., Huang C.F., Wu K.S. (2011). The association among project manager’s leadership style, teamwork and project success. IJPM.

[87] Podgórska M., Pichlak M. (2019). Analysis of project managers’ leadership competencies: Project success relation: What are the competencies of polish project leaders?. Int. J. Manag. Proj. Bus..

[88] Khan S.U.R., Long C.S., Iqbal S.M.J. (2014). Leadership competency: A tool for project success. Middle East J. Sci. Res..

[89] Oh J., Lee H., Zo H. (2019). The effect of leadership and teamwork on ISD project success. J. Comput. Inf. Syst..

[90] Nixon P., Harrington M., Parker D. (2012). Leadership performance is significant to project success or failure: A critical analysis. Int. J. Product. Perform. Manag..

[91] Liu J., Sang P., Zheng L. (2019). Exploring the influence of project manager leadership on the success of green building—Based on multi-group structural equation model. IOP Conf. Ser. Earth Environ. Sci..

[92] Kaminsky J.B. (2012). Impact of nontechnical leadership practices on it project success. J. Leadersh. Stud..

[93] Aga D.A., Noorderhaven N., Vallejo B. (2016). Transformational leadership and project success: The mediating role of team-building. IJPM.

[94] Liphadzi M., Aigbavboa C., Thwala W. (2015). Relationship between leadership styles and project success in the South Africa Construction Industry. Proced. Eng..

[95] Rogo V., Rarasati A.D., Gumuruh H. (2020). The influence of transformational leadership and soft skills on project manager for project success factors. IOP Conf. Ser. Mater. Sci. Eng..

[96] Doan T.T.T., Nguyen L.C.T., Nguyen T.D.N. (2020). Emotional intelligence and project success: The roles of transformational leadership and organizational commitment. JAFEB.

[97] Khan S.U.R., Long C.S., Iqbal S.M.J. (2015). Importance of transformational leadership in project success: A theoretical framework. Actual Probl. Econ..

[98] Iqbal S.M.J., Zaman U., Siddiqui S.H., Imran M.K. (2019). Influence of transformational leadership factors on project success. PJCSS.

[99] Caprara G.V. (2001). La Valutazione Dell’Autoefficacia: Costrutti e Strumenti.

[100] Rahmadani V.G., Schaufeli W.B., Ivanova T.Y., Osin E.N. (2019). Basic psychological need satisfaction mediates the relationship between engaging leadership and work engagement: A cross-national study. Hum. Resour. Dev. Q..

[101] Judge T.A., Colbert A.E., Ilies R. (2004). Intelligence and leadership: A quantitative review and test of theoretical propositions. J. Appl. Psychol..

[102] Gagnè M., Deci E.L. (2005). Self-determination theory and work motivation. J. Organ. Behav..

[103] Capone V., Marino L., Donizzetti A.R. (2020). The English Version of the Health Profession Communication Collective Efficacy Scale (HPCCE Scale) by Capone and Petrillo, 2012. Eur. J. Investig. Health Psychol. Educ..

[104] Capone V., Petrillo G. (2012). Costruzione e validazione della Health Profession Communication Collective Efficacy scale. Giornal. Ital. Psicol..

[105] Kaulio M.A. (2008). Project leadership in multi-project settings: Findings from a critical incident study. IJPM.

[106] Müller R., Jugdev K. (2012). Critical success factors in projects: Pinto, Slevin, and Prescott—the elucidation of project success. Int. J. Manag. Proj. Bus..

[107] Kerzner H. (2017). Project Management Metrics, KPIs, and Dashboards: A Guide to Measuring and Monitoring Project Performance.

[108] Vidal L., Marle F. (2008). Understanding project complexity: Implications on project management. Kybernetes.

[109] Lavrakas P.J. (2013). Presidential Address: Applying a Total Error Perspective for Improving Research Quality in the Social, Behavioral, and Marketing Sciences. Public Opin. Q..

[110] Capone V., Capaldo G. Organizational values and perceived pull as predictors of the university student career choice in the frame of the social cognitive career theory. Working for the greater good: Inspiring people, designing jobs and leading organizations for a more inclusive society. Proceedings of the EAWOP.

[111] Avallone F. (2011). Psicologia del Lavoro e delle Organizzazioni.

[112] Arnold J.A., Arad S., Rhoades J.A., Drasgow F. (2000). The empowering leadership questionnaire: The construction and validation of a new scale for measuring leader behaviors. J. Organ. Behav..

[113] Ricketts K.G., Bruce J.A. (2009). “Co-opetition?” Can it exist between extension and agricultural education? A study on interdisciplinary. J. Ext..

[114] Bandura A. (2006). Toward a psychology of human agency. Perspect. Psychol. Sci..

[115] LaMee N., Stedman P. (2011). Digital H: Effective leadership and decision-making. The Volunteer Management Handbook: Leadership Strategies for Success.

[116] Gist M.E., Mitchell T.R. (1992). Self-efficacy: A theoretical analysis of its determinants and malleability. Acad. Manage. Rev..

[117] Liu W., Lepak D.P., Takeuchi R., Sims H.P. (2003). Matching leadership styles with employment modes: Strategic human resource management perspective. Hum. Resour. Manag. Rev..

[118] Amundsen S., Martinsen Ø.L. (2014). Empowering leadership: Construct clarification, conceptualization, and validation of a new scale. Leadersh. Q..

[119] Davis K. (2014). Different stakeholder groups and their perceptions of project success. IJPM.

[120] Davis K. (2016). A method to measure success imensions relating to individual stakeholder groups. IJPM.

[121] Pinto J.K., Slevin D.P. (1988). Project success: Definitions and measurement techniques. Proj. Manag. J..

[122] Armstrong K.J., Laschinger H. (2006). Structural empowerment, magnet hospital characteristics, and patient safety culture. J. Nurs. Care Qual..

[123] Chen J.X., He M.Z. (2006). Project management of communication projects face conflicts. Chin. Econ. Manag..

[124] Milton A.C., Ellis L.A., Davenport T.A., Burns J.M., Hickie I.B. (2017). Comparison of Self-Reported Telephone Interviewing and Web-Based Survey Responses: Findings from the Second Australian Young and Well National Survey. JMIR Ment. Health.

[125] Roccato M. (2006). L’Inchiesta e il Sondaggio.

[126] Loewenthal K.M. (2004). An Introduction to Psychological Tests and Scales.

[127] Chasserio S., Legault M.-J. (2010). Discretionary power of project managers in knowledge-intensive firms and gender issues. Can. J. Adm. Sci..

[128] Day D.V., Fleenor J.W., Atwater L.E., Sturm R.E., McKee R.A. (2014). Advances in leader and leadership development: A review of 25 years of research and theory. Leadersh. Q..

[129] Humphrey S.E., Nahrgang J.D., Morgeson F.P. (2007). Integrating motivational, social, and contextual work design features: A meta-analytic summary and theoretical extension of the work design literature. J. Appl. Psychol..

[130] Yazici H. (2009). The role of project management maturity and organizational culture in perceived performance. Proj. Manag. J..

[131] Toivonen A., Toivonen P.U. (2014). The transformative effect of top management governance choices on project team identity and relationship with the organization. An agency and stewardship approach. Int. J. Proj. Manag..

